# Gluteal fibrosis, post-injection paralysis, and related injection practices in Uganda: a qualitative analysis

**DOI:** 10.1186/s12913-018-3711-8

**Published:** 2018-11-26

**Authors:** Kristin Alves, Christine L. Godwin, Angela Chen, Daniella Akellot, Jeffrey N. Katz, Coleen S. Sabatini

**Affiliations:** 1 Harvard Combined Orthopaedic Surgery Residency Program, 75 Francis Street, Boston, MA BTM 02115 USA; 2 FHI 360, Durham, NC USA; 3 Brigham and Women’s Hospital, Department of Orthopaedic Surgery, Division of Rheumatology, Immunology and Allergy, Harvard Medical School, Boston, MA USA; 4 CoRSU Rehabilitation Hospital, Kisubi, Uganda; 5 Department of Orthopaedic Surgery, University of California San Francisco and UCSF Benioff Children’s Hospital Oakland, Oakland, CA USA

**Keywords:** Gluteal fibrosis, Post-injection paralysis, Uganda, Injection practices, Safe injection, Pediatric musculoskeletal health, Pediatric orthopaedics, Intramuscular injection

## Abstract

**Background:**

Iatrogenic injection injury is a major cause of disability in Ugandan children. Two injuries thought to result from injection of medications into the gluteal region include post-injection paralysis (PIP) and gluteal fibrosis (GF). This study aimed to describe perceptions of local health care workers regarding risk factors, particularly injections, for development of GF and PIP. Specifically, we examine the role of injection practices in the development of these injuries by interviewing a diverse cohort of individuals working in the health sector.

**Methods:**

We conducted a qualitative study in the Kumi and Wakiso Districts of Uganda in November 2017, utilizing 68 key informant interviews with individuals working in healthcare related fields. Interviews were structured utilizing a moderator guide focusing on injection practices, gluteal fibrosis and post-injection paralysis.

**Results:**

We identified six themes regarding perceptions of the cause of GF and PIP and organized these themes into a theoretical framework. There was a consensus among the individuals working in healthcare that inadequacies of the health care delivery system may lead to inappropriate intramuscular injection practices, which are presumed to contribute to the development of GF and PIP. Poor access to medications and qualified personnel has led to the proliferation of private clinics, which are often staffed by under-trained practitioners. Misaligned economic incentives and a lack of training may also motivate practitioners to administer frequent intramuscular injections, which cost more than oral medications. A lack of regulatory enforcement enables these practices to persist. However, due to limited community awareness, patients often perceive these practitioners as appropriately trained, and the patients frequently prefer injections over alternative treatment modalities.

**Conclusion:**

This qualitative study suggests that inappropriate intramuscular injections, may arise from problems in the health care delivery system. To prevent the disability of GF and PIP, it is important to not only address the intramuscular injections practices in Uganda, but also to examine upstream deficits in access, education, and policy enforcement.

## Background

Every year an estimated 12 billion injections are performed by health care practitioners [1]. Most injections given in the developing world are provided using unsafe practices [2]. Given that many people in resource-limited countries perceive injections to be more effective than oral medications, there is a risk that injections can be used inappropriately by inadequately trained personnel [3–6]. In addition to infectious complications, disabling musculoskeletal conditions including GF and PIP have also been linked to intramuscular injections. These have been reported to occur throughout the world including Uganda, where these disabling impairments have become common health problems in children [4, 7–13]. A recently performed retrospective cohort study in Kumi, Uganda demonstrated that GF and PIP comprise over 30% of clinical hospital visits for musculoskeletal conditions and 40% of outreach visits for disabling conditions in Kumi District [14].

Clinically, PIP presents as acute flaccid paralysis and subsequent chronic acquired equinovarus foot deformities, following a gluteal injection [10, 13]. The strong temporal association between injection and onset of PIP points to an etiologic role for injections. However, the specific injection practices that may contribute to pathogenesis of PIP remain unknown. GF is a fibrotic contracture of the gluteal muscles, causing significant functional limitations While the cause has not been confirmed, GF has also been attributed to gluteal injections [7]. In one prior assessment of children with GF in Uganda all children reported histories of multiple injections, with quinine involved in 83% of the cases [9]. Given the frequency of these disabilities and the mounting evidence linking PIP and GF to injections, it is imperative to better understand injection practices in Uganda.

The primary aim of the present study was to obtain qualitative data on perceptions of individuals working in healthcare related fields regarding local injection practices and the potential role of these practices in the pathogenesis in GF and PIP. A secondary aim was to obtain qualitative data on local knowledge of risk factors for GF and PIP in Uganda. We compared views on injection practices and standards of practice among health care workers in both the northeastern rural Kumi District, a region with a high burden of GF and PIP, to those from the more central urban Wakiso District, where these disorders appear to be less prevalent [14].

## Methods

### Study design

This study utilized in-depth interviews with 68 healthcare practitioners to understand perceptions of injection practices. Interview locations included non-governmental organization run hospitals, government health centers, private clinics, public pharmacies and private drug shops in six subcounties in Kumi District and seven subcounties in Wakiso District in November 2017. The contrasting settings of the rural Kumi District and urban Wakiso District reflect areas of divergent resources and health care access. One prior study documented this divergence in resources, citing that55% of women ages 15–49 in Kumi reported serious problems in accessing health care in contrast to 13% in Wakiso [15].

Individuals were considered for inclusion in the study if they currently worked in healthcare sector, spoke English, were over 18 years of age, were currently working/residing in Kumi or Wakiso District, and were willing to provide written consent. Local experts in the health care community in Kumi and Wakiso districts helped to identify persons who met these criteria. Purposive sampling captured diversity of gender, age, professional experience, and public versus private healthcare sector involvement [16]. The key informants selected were individuals involved in the healthcare system who, due to the nature of their work, would likely have knowledge of local injection practices and who worked in a capacity that potentially exposed them to the two disabilities of interest. The informants had a range of roles and training, and included healthcare professionals such as district health officers, physicians, nurses, local practitioners, pharmacists, drug shop workers, village health workers, social workers, and government drug inspectors. “Local practitioners” refers to health care practitioners working in the private sector without formal physician training. “Village health workers” are linked to the formal health system in Uganda and provide outreach and referral into the public sector.

### Data collection procedures

The lead author (KA) conducted each interview in a closed room. Each interview was audio-recorded and facilitated by a local Ugandan research staff member (DA). All subjects answered a series of demographic questions, followed by a semi-structured interview regarding their perceptions of injection practices and underlying causes of GF and PIP.

We developed the interview guide based on formative in-country research, information regarding injection practices, GF and PIP from community leaders, and literature reviews on GF and PIP. Interview questions were tested and refined in pilot interviews in-country to ensure they could be easily understood by participants. The interviews covered the following topics: perceptions of injection practices, malaria treatment practices, knowledge or personal experience with GF and PIP, and perceived causes of GF and PIP. The semi-structured nature of the interviews allowed for discussion of these topics, as well as inclusion of participant-led perspectives and discourses. All interviews were transcribed, verbatim, for analysis.

### Ethical considerations

The study was approved by the Mildmay Uganda Research Ethics Committee, the Uganda National Council for Science and Technology, and the Partners HealthCare Institutional Review Board at Brigham and Women’s Hospital. The participants were informed about the purpose of the study and assured about the confidentiality of information they provided. Written informed consent was obtained prior to participation.

### Data analysis

Analysis of qualitative transcripts consisted of thematic content analysis employing both inductive and deductive approaches [17]. Audio recordings were transcribed verbatim, and the Dedoose web application was used for subsequent data management. Utilizing a subset of transcripts, two investigators (KA, AC) developed an initial coding framework based on emerging concepts [18]. Codes were defined as repeated concepts, and sub-codes were defined as categories within the codes. A comprehensive list of codes was established and all investigators reviewed the refined coding scheme. The coding scheme was re-assessed at regular intervals in the coding process to ensure the proper inclusion of any newly emerging ideas. Informal intercoder reliability assessments were conducted throughout the coding process to ensure consistency in the application of codes across investigators.

Two investigators (KA, AC) employed an inductive thematic analysis in which themes were extracted from the coded text to produce inferences and create a conceptual framework [19–21]. Organizing and analyzing data in this way allowed the research team to identify perceived connections between: 1) intramuscular injections practices and GF; 2) intramuscular injections practices and PIP; and 3) other possible risk factors for GF and PIP. The analysis was iterative in nature and designed to identify categories and concepts that emerged within text. Each theme was reviewed to ensure that the excerpts supported it. We sought to describe the range of views and key concerns; where considerable differences in opinions between interviewees were present, we noted and made such differences explicit. Anonymized quotations were utilized to support the interpretation of themes and to highlight the perspectives of the health care professionals and community leaders interviewed (Appendix).

## Results

### Participants

Key informants for this study were 68 individuals, including 38 men and 30 women, with ages ranging from 23 to 62 (Table 1). Participants came from 13 subcounties throughout Kumi and Wakiso District and represented 7 healthcare professions: district health officials (*n* = 3), physicians (*n* = 11), nurses (*n* = 19), pharmacists/drug shop workers (*n* = 12), social workers/community based workers (*n* = 5), village health workers (*n* = 9), and local community practitioners (*n* = 9).

**Table 1:** Participant Characteristics

	Kumi (41 total)	Wakiso (27 total)
	No. (%)	No. (%)
Gender		
Female	18 (43.9)	12 (44.4)
Male	23 (56.1)	15 (55.6)
Age		
18–29	12 (29.3)	7 (25.9)
30–44	20 (48.8)	13 (48.1)
45–60	8 (19.5)	7 (25.9)
60+	1 (2.4)	0 (0)
Profession		
District Health Official	2 (4.9)	1 (3.7)
Physician	8 (19.5)	3 (11.1)
Nurse	12 (29.3)	7 (25.9)
Pharmacist/Drug Shop worker	7 (17.1)	5 (18.5)
Local Practitioner	6 (14.6)	3 (11.1)
Village Health Worker	3 (7.3)	6 (22.2)
Other	3 (7.3)	2 (7.4)
Professional Sector		
Private	20 (48.8)	12 (44.4)
Public	21 (51.2)	15 (55.6)

All practitioners were asked if they had personally seen cases of GF and/or PIP. While all 41 participants in Kumi District had seen both GF and PIP, only 33% (9/27) of Wakiso participants had first-hand experience with GF and only 56% (15/27) with PIP. For those Wakiso participants who did have first-hand experience, most had not seen the conditions in a long time or saw PIP and GF in previous work outside of Wakiso.

### Thematic analysis – Overview

**Fig. 1:**
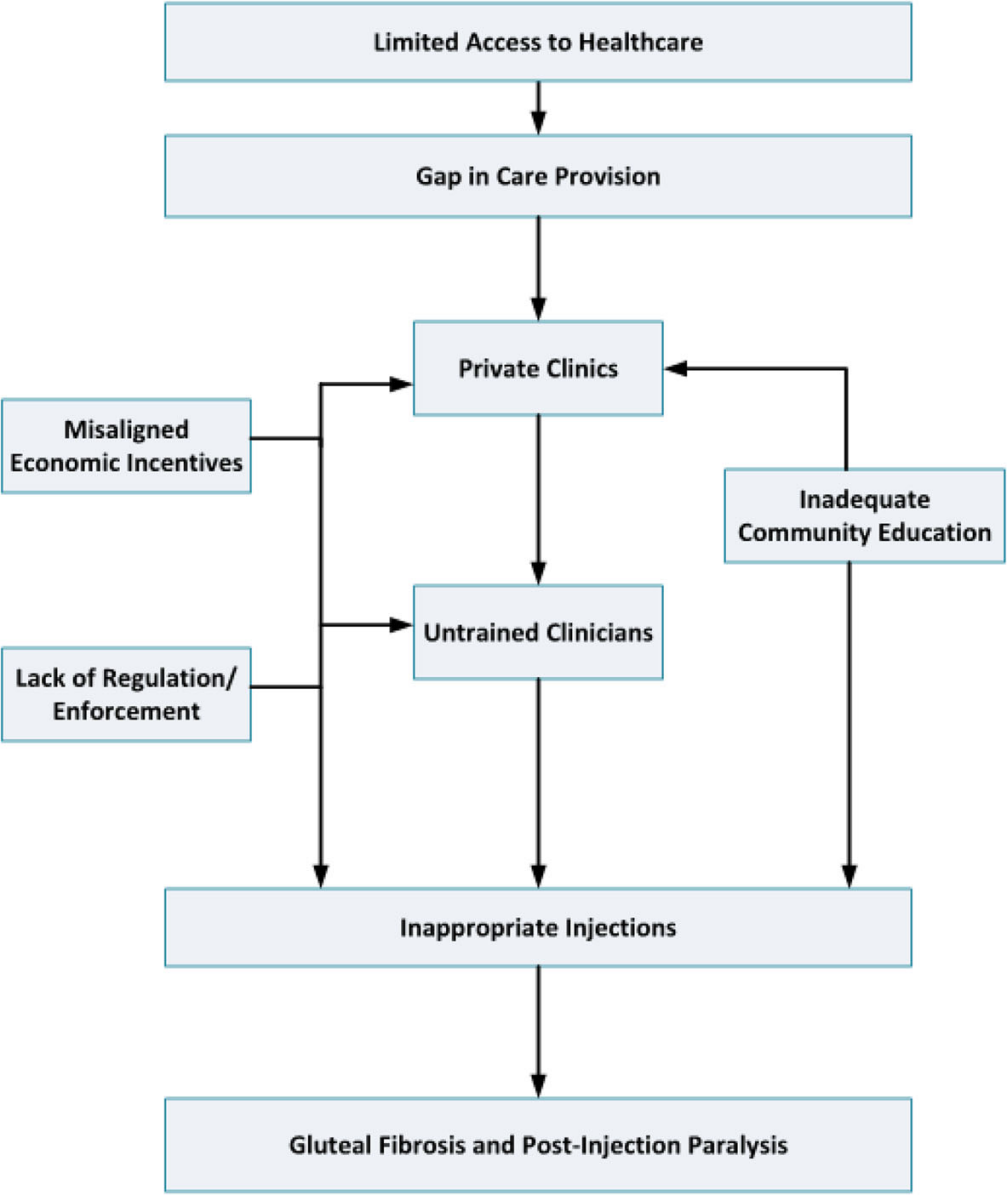
Theoretical Framework for systems issues contributing to cases of GF and PIP

We identified six themes regarding the perceptions of the underlying cause of GF and PIP and we organized these themes into a theoretical framework (Fig. 1). Across districts, professions and gender, participants consistently reported a perception that GF and PIP were the result of intramuscular (IM) injections. Further, the participants attributed administration of these injections to challenges in the health care delivery system. Specifically, a lack of accessibility to medications and qualified personnel resulted in a gap in care that has been increasingly filled by private clinics. These clinics – often staffed by untrained personnel and motivated by misaligned economic incentives, with increased pay for injections in comparison to oral medications, and a lax regulatory environment – seem to be a common site of inappropriate administration of intramuscular injections. Furthermore, due to a lack of community education about proper medical practices, these practitioners are perceived as being appropriately trained and patients often prefer injections over other treatment modalities. In the following sections, we present each of these themes in detail and discuss the supporting data.

#### Inappropriate intramuscular injections are the perceived cause of GF & PIP

Throughout the interviews, participants consistently reported injections as the cause of GF and PIP. Potential drugs reported as administered IM in the community included quinine, penicillin, and gentamycin, with a variety of others noted much less frequently. Participants reported that quinine was most commonly involved in the development of GF.

*“[Gluteal intramuscular injections given frequently to children include] …quinine, gentamycin and penicillin.” (Kumi, Public Nurse)*

While participants from both districts reported that diclofenac, a drug used to treat pain, was primarily administered intramuscularly, many reported their belief that use of diclofenac was unrelated to the development of GF and PIP. Several clinicians reported knowledge of children receiving diclofenac with no known complications.

*“The only thing I’ve seen being given in the buttocks [in Wakiso] of late is diclofenac. I’ve not seen anyone with gluteal fibrosis.” (Wakiso, Private Physician).*

The most frequent responses regarding the ways in which intramuscular injections may lead to health issues suggested that 1) PIP occurs when injections are administered at the wrong site, close to the sciatic nerve and 2) GF develops due to infection stemming from poor sterility practices or necrosis secondary to toxic medications.

*“There is no specific drug, the cause is that the drug is injected by nonprofessional injecting it in a wrong site which brings paralysis.” (Wakiso, Public Nurse).*

Regarding necrosis causing GF, participants cited incorrect frequency or dilution during administration in addition to the toxicity of the drug itself. Specifically, the frequent dosing of quinine and penicillin injections subjects children to an excessive number of injections for a single episode of illness.

*“They don’t dilute. It is very toxic to the muscle. In the healing process of the muscle there is fibrosis.” (Kumi, Public District Officer).*

While the majority of participants felt injections were the cause of GF and PIP, a few participants suggested that other factors, in conjunction with injections, might impact the development of GF and PIP in children. Some suggested that the geographic distribution of GF and PIP in Uganda might be explained by a genetic predisposition of the ethnic groups that populate the most affected regions.

*“You see, not everyone [who has intramuscular injections] gets [gluteal fibrosis]. Maybe it’s something that these particular children have, either their healing is compromised…People who get keloids and have hypertrophic scars that run in families. The individuals may have a particular risk factors that make them prone to these disabilities.” (Wakiso, Private Physician).*

Participants highlighted eastern Uganda as a region where disabilities related to GF and PIP are prevalent.

*“Yes I have heard of gluteal fibrosis, in the northeast.” (Wakiso, Public Village Health Worker).*

Other participants expressed concern that GF and PIP may occur in regions outside of the northeast, but may be misdiagnosed due to poor knowledge of the conditions.

*“The problem is many people don’t know it. Like I also thought then that it was probably to do with exaggerated femoral anteversion or retroversion or something like that…Some of them think it may be a form of muscular dystrophy. Some of them think it’s cerebral palsy. It’s being misdiagnosed. The problem is many people do not know about it.” (Kumi, Private Physician).*

#### Role of accessibility

While all participants reported a belief that GF and PIP were directly caused by injections, many went on to describe the system-level factors contributing to the inappropriate injection practices. Participants in both districts described persistent problems with government-run public health facilities, including frequent shortages of drugs and a limited number of trained healthcare workers. These issues, which potential patients consistently encounter at government facilities, can decrease confidence in the public health system and encourage patients to seek care at private facilities.

*“The number of personnel that are trained is not enough. There is not a big number, but there are a lot of people who need care.” (Kumi, Public Pharmacy Worker).*

#### Filling the gap: Private clinics

Participants cited that these gaps in care are being filled by private clinics which are often staffed by untrained professionals. Private clinics visited by the research team were run by nursing assistants, lab technicians, and on-the-job trained staff who discussed how care provision for their communities included intramuscular injections. Each of these practitioners described short periods of informal training without nursing or medical degrees. Per the interviewees, these private clinics are often preferred by patients as they are more accessible in terms of distance, cost and time.

*“Most of these [private] facilities are run by nursing assistants or somebody who maybe did some lab work or somebody who has ever worked at a hospital…They start up clinics, and they give all those kind of medications.” (Wakiso, Private Nurse).*

#### Untrained clinicians

The government employed healthcare sector interviewees contend that the untrained or unqualified practitioners working in the private clinics do not have the knowledge to administer the correct medications nor skills to deliver appropriate intramuscular and intravenous medications.

*“Some of them might not knowing even the sites [or sterile techniques], because they are not qualified personnel.” (Kumi, Public Nurse).*

In addition, the subset of government employed participants acknowledge that the private clinic workers do not have access to continuing medical education to learn current treatment practices because they are not recognized by the government.

*“I have attended workshops before when I was still working at the government hospital; I was working there and could attend them. Private clinicians are not invited.” (Wakiso, Private Local Practitioner).*

#### Misaligned economic incentives

The government employed participants described the private clinics as profit-run businesses that prefer to hire lower-cost, untrained clinicians. Additionally, the government and privately employed participants pointed out that the private business model motivates these clinics to provide faster care with “better” customer service.

*“In those private clinics, they have better customer care unlike in public hospitals or facilities where there are lines and you can go and sit there for hours. In the private clinic they know this is a business. They know if they don’t give good customer care they lose.” (Wakiso, Public Community-Based Rehabilitation Worker).*

The pricing of the IM injections may also contribute to their frequent use. The injections are more profitable than oral medications, which incentivizes private providers to administer them, but cost less than the IV medications, which are often too expensive and time-intensive for patients and their families.

*“…people they can sell [injections] to get more money than with oral medications.” (Kumi, Private Local Practitioner).*

#### Inadequate community education

The frequent use of injections is further encouraged by the lack of education of patients and families regarding practitioners’ qualifications and proper treatment methods. Patients are largely unable to differentiate between the public health centers and the private clinics and are unaware of the lack of training of the practitioners in the private clinics. Furthermore, many patients also associate quality with cost of care, believing that the pricier private clinics provide higher quality of care.

*“Then those people don’t have a lot of knowledge to know that this one is qualified or not. They think anyone with a white gown is qualified.” (Wakiso, Public Nurse).*

Moreover, interviewees agreed that the community has developed misconceptions that intramuscular injections are superior to other medical treatments. Thus, families demand injections for their greater perceived efficacy.

*“They prefer an injection. For most of them, they think the injections act faster. They think injections get to the bloodstream faster than tablets.” (Kumi, Public Physician).*

#### Lack of necessary regulation/enforcement

There was consensus that the proliferation of private clinics, staffed by untrained professionals, has gone unchecked due to a lack of regulation and policy enforcement. Interviewees voiced concerns regarding the lack of district health officials to enforce policies, resulting in poor monitoring of rural areas.

*“Well the laws are there, but the enforcement is poor.” (Kumi, Public District Officer).*

## Discussion

This study elicited information from 68 participants from diverse health care professions and job types regarding injection practices and potential risk factors for GF and PIP in Uganda. The main theme was the perception that gluteal IM injection practices are the primary cause of GF and PIP. While prior assessments of the safety of IM injection practices have focused on rates of transmission of bloodborne viral pathogens, this study demonstrates the critical need to consider the risk of musculoskeletal injury following IM injection, in developing policies [4, 5, 22].

The interviews documented inadequate training of personnel giving injections, regardless of medication delivered, in the incorrect gluteal site as leading to PIP. This finding is supported by a prior study in Uganda demonstrating that drugs injected prior to PIP included quinine, antibiotics, antipyretics and chloroquine [13]. Unfortunately, sciatic nerve injury due to inappropriate training remains a persistent and global problem with a need for improvement in education of site selection [23–25].

Regarding the pathogenesis of GF, informants perceived that such practices may cause infection and additionally noted that injection of specific drugs such as quinine, penicillin and gentamycin may cause necrosis. The consensus among participants interviewed suggests a focus on sterility and toxicity with any future research attempting to determine the specific pathogenesis of GF.

Although IM injection practices might be the direct cause of GF and PIP, as the theoretical framework illustrates, larger health care practice concerns appear to encourage greater use of the inappropriate injection practices. As in other resource-limited countries, these injections are reported to be administered by a variety of health care practitioners with differing qualifications and training [4, 5]. The consensus of interviewees was that unqualified practitioners are administering the IM injections in private clinics in rural areas. This corroborates the findings of previous studies demonstrating that most injections delivered in Uganda were given by private non-formal providers [22, 26]. The interviewees contend that these private clinics primarily exist in rural areas, including northeastern Uganda. This contention corresponds with the findings of this study, in which 100% of practitioners in rural northeastern Kumi district had seen cases of GF and PIP, while 44 and 67% of practitioners in the urban central Wakiso district had seen patients with GF and PIP, respectively.

The existence of the private clinics with under-qualified or untrained staff stems from problems with access, education of the community, misaligned economic incentives and lack of policy enforcement. An informal health sector developed in response to the gap in the provision of public health care, and the private clinics have addressed the excess demand for care by employing a profit-driven business model which involves the hiring of a lower-cost untrained workforce. Furthermore, the public is largely unaware of the difference in qualifications. Similar development of an informal, underqualified health sector has occurred in other resource-limited settings [4]. Compounding these issues is a lack of effective monitoring and regulation, which allows this informal sector to exist.

Knowledge of these contributing factors cited by practitioners can be utilized to address upstream causes of GF and PIP, which may have a greater impact for the population. Interventions to address the current misuse of injections include improvement in accessibility/health care delivery, community education on medical treatments and qualifications of clinicians, training of providers with potential for task shifting and education for the informal sector, and regulatory actions to support policy (Table 2).

**Table 2:** Proposed interventions for improvement of unsafe injection practices

Intervention	Examples
Improvement Accessibility/Healthcare Delivery	Work with district officers to ensure adequate drug stocks of critically important medications Increasing trained/qualified workforce
Regulation/Policy Enforcement	Improve control of the sale and use of injection equipment Enforcement of policy
Education: Providers	Carry out continuing medical education programs for all providers on safe injection practices and treatment guidelines Train non-formal providers in safe practices
Education: Community	Inform public on potential harm of intramuscular injections and adequacy of oral medications Sensitize community to qualifications of providers

Limitations for this qualitative analysis include the focus on two districts in Uganda, which may exclude potentially relevant practices occurring outside of this area. In addition, the study’s findings are limited to the perceptions of the healthcare community. This study was designed to take place where the disorders are seen or reported frequently to provide information-rich, exploratory, and hypothesis-generating data. Given that IM injections in low-resource countries are a common and often unnecessary practice, it is essential that injection practices are addressed [4, 5, 22].

## Conclusion

The findings of this study suggest that GF and PIP are iatrogenic complications of inappropriate intramuscular injections secondary to upstream healthcare delivery system limitations. To prevent these musculoskeletal disorders and the resulting disability, it is important to address upstream deficits in accessibility, education, and policy enforcement. The findings from this study can inform practical interventions that are urgently needed to address healthcare delivery in Uganda and thus prevent further cases of GF and PIP.

## Appendix

**Table 3:** Thematic analysis results with supporting quotations from healthcare and community leader interviews

Kumi	Wakiso
Consensus: Inappropriate intramuscular injections are the perceived cause of GF & PIP	
• “The outcome has always been attributed to quinine. The mother's report that on the onset of injection, the child gets paralyzed. The leg will just be stiff. Others get gluteal fibrosis, failing to squat on knee bend.” (Kumi, Public Village Health Worker) • “[Gluteal intramuscular injections given frequently to children include] …quinine, gentamycin and penicillin.” (Kumi, Public Nurse) • “[there’s an] epidemic, of intramuscular injections….And quinine itself was toxic to the muscles. Some of them ended up with a fibrosis much later because quinine was probably not properly diluted. Not everyone was giving the correct dilution of quinine for the muscles so I think there was quinine toxicity in the muscles that led to the healing by fibrosis.” (Kumi, Private Physician) • “When penicillin is given IM to children, it causes fibrosis. And as time goes on sometimes the child gets an abscess…If they hit the wrong site then the child can develop paralysis.” (Kumi, Public Nurse) • “Now, if they inject a kid in the buttocks in the wrong position it will automatically cause that nervous effect in the buttocks and it brings that paralysis.” (Kumi, Private Nurse) • “For the paralysis it is mostly due to an injection that is causing a swelling that compresses the nerve or an injection directly into the nerve that can cause a paralysis. So, any medicine injected in the wrong place which targets the nerve can give you a paralysis. What you’re giving doesn’t matter for the injection… In terms of the fibrosis, there are a few documented agents or drugs that can cause tissue necrosis. Something which is less observed or something that is bound to cause after injection can in the long run cause fibrosis. It is toxic.” (Kumi, Public Pharmacy Worker) • “A number of [children with gluteal fibrosis] start by getting an abscess. And then that abscess will not be managed properly. In the healing process there is fibrosis.” (Kumi, Public District Officer) • “So we also need to look at what effect does it have with the number of different injections. Maybe it is a cumulative effect. Or there are trigger factors, that when you keep pricking the buttock it triggers something.” (Kumi, Public District Officer) • “…in the rural setting, in those clinics, some of them rarely have the weighing scale…It is guesswork. The weight is guessed….Maybe I have too high a dose….we have children who have received injections. Could be the child has fallen sick 3 times in just two weeks. So when the child goes there they get injections each time.” (Kumi, Public Nurse) • “I strongly feel that there’s a causative agent but also host factors must be responsible because in this region, with this genetic stock of people, [these disabilities] are quite common around Teso region. Because I’ve worked in western Uganda, they’ve also given injections, but we haven’t seen these injections injuries…So, I think [injections are] a precipitating factor, but there must be host factors…The type of collagen maybe we have here might be different from the collagen other people have because in addition to the gluteal fibrosis, we also see a lot of old men here with urethral stricture disease, urethral fibrosis…So, there might be a genetic component. Maybe the collagen.” (Kumi, Private Physician) • “The problem is many people don’t know it. Like I also thought then that it was probably to do with exaggerated femoral anteversion or retroversion or something like that…Some of them think it may be a form of muscular dystrophy. Some of them think it’s cerebral palsy. It’s being misdiagnosed. The problem is many people do not know about it.” (Kumi, Private Physician)	• “The only thing I've seen being given in the buttocks [in Wakiso] of late is diclofenac. I've not seen anyone with gluteal fibrosis.” (Wakiso, Private Physician) • “There is no specific drug, the cause is that the drug is injected by nonprofessional injecting it in a wrong site which brings paralysis.” (Wakiso, Public Nurse) • “They give an injection and it’s not sterile. Sometimes I might find some kind of infection after injection, and then the formation of fibrosis.” (Wakiso, Private Local Practitioner) • “They don’t dilute. It is very toxic to the muscle. In the healing process of the muscle there is fibrosis.” (Kumi, Public District Officer) • “Even if you are injected from a normal position, you’re getting 3 - 4 injections in 24 hours times 5 days…” (Wakiso, Public Nurse) • “You see, not everyone [who has intramuscular injections] gets [gluteal fibrosis]. Maybe it's something that these particular children have, either their healing is compromised…People who get keloids and have hypertrophic scars that run in families. The individuals may have a particular risk factors that make them prone to these disabilities.” (Wakiso, Private Physician) • “The few patients I’ve seen…either they’re coming from the north or the east. I haven’t seen any patient from Wakiso.” (Wakiso, Private Nurse) • “Yes I have heard of gluteal fibrosis, in the northeast.” (Wakiso, Public Village Health Worker)
Theme 1: There is limited access to healthcare	
• “The number of personnel that are trained is not enough. There is not a big number, but there are a lot of people who need care.” (Kumi, Public Pharmacy Worker) • “When you go to the hospital, you go in the morning and you come back in the evening. It is a long distance…Most of them don’t have that time. You might even come back without treatment.” (Kumi, Private Drug Shop Worker)	• “It is a huge population so [drug stockouts] commonly happen…[The private clinics] purchase when they need. But for us, we wait. The government hospitals have to wait for the national medical council to deliver.” (Wakiso, Public Physician)
Theme 2: Gap in care provision has led to frequent use of private clinics	
• “You go to the health unit and they say there are no drugs, go and buy. So people tend to fill in that gap. So instead of you going very far, why not just go [to the private clinic]. And then when you go there you are given things you want.” (Kumi, Public District Officer) • “In the villages we don’t have trained health workers reaching to those hard to reach areas, but there are people who need treatment…So what do they do, they improvise, they find somebody that can give [them treatment]…You see our country is still primarily rural. There are so many places that are hard to reach…And you know whenever there is a gap there is always people that will fill the gap. So we have a number of private clinics in those hard to reach areas. They’re actually handled and open by untrained persons.” (Kumi, Public Pharmacy Worker) • “So there’s a problem with the health care service provision. There is no qualified doctor to give the right route of administration in the rural setting. So these quacks get ahold of this drug and they give it…People go to the [private] clinics because the distance.” (Kumi, Private Physician)	• “Most of these [private] facilities are run by nursing assistants or somebody who maybe did some lab work or somebody who has ever worked at a hospital…They start up clinics, and they give all those kind of medications.” (Wakiso, Private Nurse)
Theme 3: The private clinics predominately utilize unqualified clinicians who are not trained to give IM injections	
• “In the rural settings…people have gone ahead to open up [private] clinics, and they are not trained in the techniques on how to inject. And most of these people end up getting those problems when they are injected in the clinics.” (Kumi, Public Nurse) • “There are people masquerading to be nurses and doctors in the villages who are going to inject. They do it in the wrong sites and find that kids end up with a problem.” (Kumi, Public Physician) • “Some of them might not knowing even the sites [or sterile techniques], because they are not qualified personnel.” (Kumi, Public Nurse) • “They say we are failing to get the vein. So let’s inject [the quinine]. But the initial right route is through IV. In the private practice, not all people are trained, there is a lack of knowledge that they’re supposed to give IV and some are not trained on how to do it.” (Kumi, Public District Officer)	• “They are not trained enough. Some of these nurses, they have difficulty finding the vein and sometimes they may learn how to do it, but not enough training still. It is easier to give an injection. You just prick. While finding a vein is difficult.” (Wakiso, Public Village Health Worker) • “We have a very serious gap. [We are] good at developing the policies, launching them, but not dissemination…They are disseminating the policies mainly to public facilities, leaving these people from the private facilities without continued education.” (Wakiso, Public District Officer) • “I have attended workshops before when I was still working at the government hospital; I was working there and could attend them. Private clinicians are not invited.” (Wakiso, Private Local Practitioner)
Theme 4: The private clinics have misaligned economic incentives increasing utilization of IM injections	
• “…you cannot place a doctor in the village. How will you pay him, and do you have enough people to come to that clinic? You resort to taking these ordinary untrained people, so long as you have showed him how to treat then they will work there. Of course, the outcome will be the patient to suffer because the person is not actually trained.” (Kumi, Public Social Worker) • “…people they can sell [injections] to get more money than with oral medications.” (Kumi, Private Local Practitioner) • “Most of them have an understanding that when you’re given the injection in the buttock it’s faster [than intravenous]. So when they are giving injections, the other one is doing a business and gets more. They give them what they want. They get more business, more money, more injections.” (Kumi, Public Nurse)	• “In those private clinics, they have better customer care unlike in public hospitals or facilities where there are lines and you can go and sit there for hours. In the private clinic they know this is a business. They know if they don’t give good customer care they lose.” (Wakiso, Public Community-Based Rehabilitation Worker)
Theme 5: There is inadequate community education regarding appropriate quality healthcare delivery	
• “The people in the village cannot distinguish who is a qualified health person and who is not. So they just go to where they think there is a service.” (Kumi, Public District Officer) • “They prefer an injection. For most of them, they think the injections act faster. They think injections get to the bloodstream faster than tablets.” (Kumi, Public Physician)	• “Then those people don’t have a lot of knowledge to know that this one is qualified or not. They think anyone with a white gown is qualified.” (Wakiso, Public Nurse) • “People associate quality with money…Where you pay money, the quality must be better than where it is free, not knowing that the government is paying.” (Wakiso, Public District Officer) • “We need continue the campaign on the dangers of giving and receiving injections. Because in most cases the person that comes in thinking if you don’t give me an injection I won’t get well.” (Wakiso, Public Community-Based Rehabilitation Worker)
Theme 6: There is a lack of necessary regulation/enforcement for injection practices	
• “Well the laws are there, but the enforcement is poor.” (Kumi, Public District Officer) • “The officer tries to monitor. But there are many of them [private clinics].” (Kumi, Public Nurse)	• “The chance of getting someone trying to do something wrong within Kampala is lower [because] it is easier to report them. When they’re far deep down in the village, the practices there are least monitored.” (Wakiso, Private Drug Shop Worker) • “ In the urban areas they fear inspection, but in the villages, rarely someone goes to check on these facilities. If anybody will check, [the clinic workers] will be alerted, people coming around, close their facility…The monitoring mechanism is very poor. We’ve got so many health centers in Uganda, private and the public, but really you find no one is following up on the clinicians or the nurses. No one is doing that routine follow-up to see how are they doing their work; are they doing the right things or not?” (Wakiso, Private Community-Based Worker)

## Abbreviations

GF: Gluteal Fibrosis
IM: Intramuscular
PIP: Post-injection paralysis
